# Volcanically Triggered Ocean Warming Near the Antarctic Peninsula

**DOI:** 10.1038/s41598-019-45190-3

**Published:** 2019-07-01

**Authors:** L. S. Verona, I. Wainer, S. Stevenson

**Affiliations:** 1 Instituto Oceanográfico, Universidade de São Paulo (IO/USP), São Paulo, SP Brazil; 2 Bren School of Environmental Science & Management, University of California, Santa Barbara, CA USA

**Keywords:** Physical oceanography, Physical oceanography, Palaeoclimate

## Abstract

Explosive volcanic eruptions are the largest non-anthropogenic perturbations for Earth’s climate, because of the injection of sulfate aerosols into the stratosphere. This causes significant radiation imbalances, resulting in surface cooling for most of the globe. However, despite its crucial importance for Antarctic ice sheet mass balance, the response of the Southern Ocean to eruptions has yet to be understood. After the eruption of Mt. Pinatubo in 1991, much of the Southern Ocean cooled; however, off the Antarctic Peninsula a warming of up to 0.8 °C is found in the observations. To understand the physical mechanisms associated with this counter-intuitive response, we combine observational analysis from the Mt. Pinatubo eruption with the Last Millennium Ensemble (850–1850) conducted with the Community Earth System Model. These results show not only that the observed warming off the Peninsula following the Mt. Pinatubo eruption is consistent with the forced response to low-latitude eruptions but further, that this warming is a response to roughly 16% weakening of subsurface Weddell Gyre outflow. These changes are triggered by a southward shift of the Southern Hemisphere polar westerlies (∼2°latitude). Our results suggest that warming induced by future volcanic eruptions may further enhance the vulnerability of the ice shelves off the Antarctic Peninsula.

## Introduction

Volcanic eruptions are important elements of the climate system, driving transient changes to global climate on interannual to decadal timescales^1–3^. Large explosive eruptions are capable of injecting sulfate aerosols into the stratosphere, which are responsible for reducing incoming shortwave radiation^1,3^. The global response is an overall cooling of Earth’s surface^1,4–7^. The eruption of Mt. Pinatubo in 1991 was the largest eruption of the 20^*th*^ century^1,8–13^. It caused global sea surface cooling of 0.3–0.4 °C^4,5^ with impacts on weather and hydroclimate throughout the world^14^. Nonetheless, its size is comparably small relative to the eruptions of the past millennium, before 1850 where anthropogenically driven warming was insignificant^15^. Thus, understanding the range of regional impacts associated with past large volcanic eruptions is crucial for assessing the impacts of potential future eruptions. This is particularly relevant given the potential for future anthropogenic warming to modify the impacts of volcanic eruptions^16,17^.

The Southern Ocean is a vulnerable region to climate change, in part because of the presence of floating ice shelves around most of Antarctica’s coastline^18^. In addition, it is the site of dense water formation (i.e. Antarctic Bottom Water) and unique ecosystem communities^19^. The Antarctic Peninsula ice shelves and ocean near it deserve special attention because of ongoing changes to ecosystems and ocean-atmosphere-cryosphere dynamics due to climate change^20^. The Weddell Sea region off the Antarctic Peninsula has been widely impacted by recent permanent ice-shelve collapse^21,22^ and thinning (i.e. Larsen Ice Shelf)^23–25^. Antarctic Peninsula ice shelves are highly susceptible to changes in ocean circulation, as observed in the Larsen C ice loss through basal melting^23,26^ and intrusion of warm ocean waters^18,27^. After a large eruption sea-ice extension responds more to ocean temperature and circulation changes than to the direct radiative forcing^2,7,28^. Climate simulations have shown that the eruption aftermath in Antarctica is an initial sea-ice expansion followed by its contraction^29^ linked primarily to the strengthening of the Southern Hemispheres stratospheric polar vortex and a chain of regional feedbacks. The connectivity between large-scale dynamics and local processes after an eruption in the Southern Ocean highlights several relevant features that lack of studies^29^, as ocean temperature exchange and regional polar interaction between ocean-atmosphere and sea-ice.

Climate models can provide valuable insight into the physical mechanisms driving responses to volcanic eruptions. The use of ensembles is particularly valuable, due to the large extent of internal climate variability which can mask the forced response to eruptions^30–35^. Here we make use of the Community Earth System Model Last Millennium Ensemble (CESM-LME^33^), which contains multiple realizations of the climate over the 850–2005 period. This allows us to examine both the effects of the 1991 Mt. Pinatubo eruption individually, and the potential impacts of larger Last Millennium (LM, 850–1850) eruptions more broadly, to identify common physical mechanisms in the Southern Ocean response to eruptions.

## The Modern Eruption of Mt. Pinatubo

**Figure 1:**
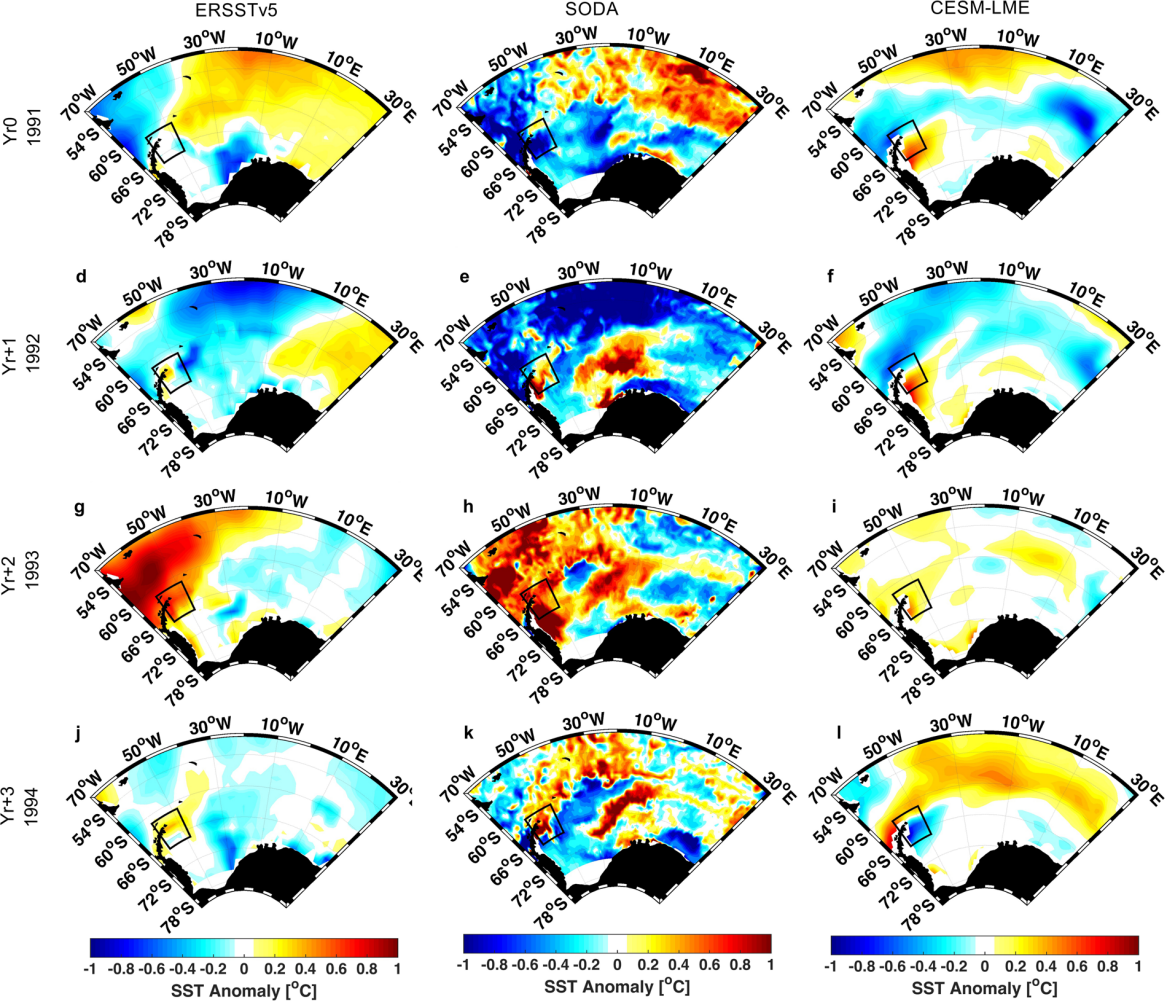
DJF SST [°C] after Mt. Pinatubo eruption. Left-hand column for ERSSTv5 **[(a),(d),(g),(j)]**, central column for SODA **[(b),(e),(h),(k)]** and right-hand column for CESM-LME **[(c),(f),(i),(l)]**. Each row represents different years from 1991 (Yr0, the eruption year) to 1994 (Yr + 3). The delimited area marks the region off Antarctic Peninsula (60°-48°W, 61°-67°S).

The impact of the Mt. Pinatubo eruption on the Atlantic sector of the Southern Ocean is investigated using observations (Extended Reconstructed Sea Surface Temperature version 5 - ERSSTv5^30^ and Hadley Centre Sea Ice and Sea Surface Temperature - HadISST^31^) for sea surface temperature (SST) and oceanic reanalysis (Simple Ocean Data Assimilation version 3.4.1 - SODA3^32,36^ and Ocean Reanalysis System 4 - ORAS4^11^) for SST and wind stress. We combine the observational results with the output from CESM-LME^33^, which contains 15 realizations (10 runs considering all natural and anthropogenic forcing factors and 5 volcanic-only runs) of the 850–2005 period including the effects of volcanism. The use of 15 ensemble members allows an improved estimate of the uncertainty due to internal variability^37^. All results shown here are restricted to the austral summer (DJF, see Methods for definition), since the volcanic response was identified to be larger during this season^4^ and there is less presence of ice in the Weddell Sea. For the Mt. Pinatubo analysis the anomalies of SST and related fields are calculated relative to the 1980–2005 climatological mean, which contains the anthropogenic signal^38,39^. The time series are de-trended, over the same period, to minimize the effects of long-term positive trend of increased greenhouse gases and ozone depletion forcing. The evolution of the observed SST spatial pattern for the Mt. Pinatubo eruption from the year of the eruption (Yr0, 1991) to Yr + 3 (1994) is shown in Fig. 1 and Supplementary Fig. S1.

During the year of eruption (Yr0, 1991) the SST anomaly presents similar pattern in all observational data sets (Fig. 1a,b and Supplementary Fig. S1). There is a cold anomaly of about −1 °C in the western region and in the Weddell Sea. At the same time that a warm anomaly is present in the northeast region (mostly north of 66°S) ranging from 0.3 °C (ERSSTv5 and HadISST) to 1 °C (SODA and ORAs4). On the other hand, the model ensemble average from CESM-LME (Fig. 1c) shows a cold anomaly (−0.6 °C) between 54°S and 66°S and a warm anomaly off the Antarctic Peninsula in the Weddell Sea of 0.6 °C. In the year following the eruption (Yr + 1, 1992, Fig. 1d,e,f and Supplementary Fig. S1) the general pattern of all data sets is similar. There is negative anomaly in the SST, north of 60°S it reaches −1 °C. The warm anomaly distribution is spatially smaller than Yr0, it occurs in the eastern sector (~ 0.2 °C), but also in a small region off the Antarctic Peninsula. ERSSTv5, HadISST and ORAs4 show a smaller warming of ∼0.3 °C; SODA and CESM-LME presents higher values of about 0.8 °C.

At Yr + 2 (1993, Fig. 1g,h and Supplementary Fig. S1), the warming in the Weddell Sea reaches its largest value (0.8 °C), accompanied by a warming north of the Antarctic Peninsula for all observational data. For CESM-LME ensemble average the warm anomaly has decreased, off the Antarctic Peninsula to 0.4 °C, and northward of the Peninsula to 0.2 °C. The cold anomaly has decreased for all data sets compared to the previous year. During Yr + 3 (1994, Fig. 1j,k and Supplementary Fig. S1) the anomaly north of the Antarctic Peninsula almost disappears and the warm anomaly in the Weddell Sea is still present for all data sets. However, CESM-LME (Fig. 1l) shows the opposite pattern.

Results from all observational data sets show an overall similar pattern, albeit differences in anomalies magnitude. The same pattern is somehow present in the CESM-LME ensemble average; however, all anomalies are underestimated and spatially smoothed. CESM-LME simulation results contrasts from the observations at Yr0. In the observational data sets the warming response off the Antarctic Peninsula starts in the first year after the eruption (Yr + 1, 1992) and extends to Yr + 2 (1993), while in CESM-LME it appears in the year of the eruption (1991) and persists until 1993 (Yr0 to Yr + 2). The average of the CESM-LME volcanic-only ensemble members (Supplementary Fig. S1) shows the warming response near the Peninsula only from Yr + 1 to Yr + 2 (1992 to 1993). Therefore, the warming seen at Yr0 (1991) does not seem to be related to the volcanic forcing. It should be noted that although there is warming off the Antarctic Peninsula post eruption for all data sets, the warming is not unique to this region, particularly in the observational data sets. This could indicate an issue with the simulated response to forcing in the CESM-LME, but there could also an important contribution from internal climate variability^33^.

**Figure 2:**
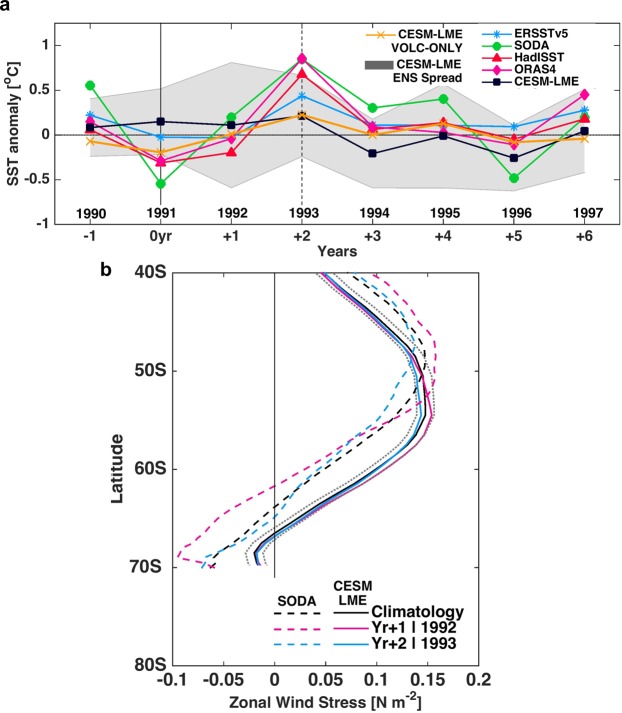
DJF SST anomaly time series and zonal averaged wind stress changes after Mt. Pinatubo eruption. (**a**) SST anomaly [°C] time series for the Antarctic Peninsula region (60°-48°W,61°-67°S). (**b**) Zonal averaged wind stress [N m^−2^] for the South Atlantic Ocean (70°W-30°E, 40°-70°S). The dashed line represents SODA reanalysis and the continuous line CESM-LME. The grey dotted lines represent CESM-LME ensemble spread. Black lines are the climatology mean for 1980–2005.

We focus here on the Antarctic Peninsula due to its potential in contributing towards future sea level rise through ice shelf failure^21,22^. To help understand the evolution of the detected positive SST anomaly in this region, we explore the time series for a specified region off the Antarctic Peninsula (60°-48°W, 61°-67°S, area marked in Fig. 1). Results (Fig. 2a) show that the largest averaged signal for this region after the eruption is a sea surface warming during the second subsequent year (Yr + 2, 1993): CESM-LME (0.2 ± 0.45 °C), ERSSTv5 (0.4 °C), SODA and ORAS4 (0.85 °C) and HadISST (0.7 °C). CESM-LME presents the smaller warming off the Antarctic Peninsula because of the ensemble average that showed large variability (±0.45 °C). Analysis of CESM-LME simulation results allows us to separate the volcanic signal from other forcings. The volcanic-only ensemble average shows the same positive anomaly near the Antarctic Peninsula at Yr + 2 (1993) of ∼0.2 °C (orange line in Fig. 2a), which attributes this warming to the volcanic forcing.

Zonal wind stress changes obtained with the results of SODA and CESM-LME reveal similar behavior (Fig. 2b). After the Mt. Pinatubo eruption, at Yr + 1, the prevailing westerlies have increased in magnitude (4 to 10% from the climatological mean) and its southern extension shifted southward (Supplementary Fig. S2). The maximum wind stress position is determined by the zonal wind stress weighted mean latitude based on the latitude where it is greater than 50% of its maximum^40^. During the first year after the eruption (Yr + 1, 1992) the southward shift is of ~0.54° latitude in CESM-LME (magenta continuous line in Fig. 2b) and ~1.30° latitude in SODA (magenta dotted line in Fig. 2b). By 1993 (Yr + 2, blue lines in Fig. 2b) the averaged zonal wind stress becomes smaller than the climatological mean (black lines). A southward shift*/*expansion and straightening^29^ of the westerly wind system is related to poleward shifts in the Antarctic Circumpolar Current (ACC)^41–44^. A shift of the westerlies can draw more water from deeper layers to the surface around Antarctica^45^ and modify subsurface temperature^46,47^.

The aftermath of a volcanic event is an overall decrease in the SST^4,5,7^, which acts to offset the anthropogenic warming^38,48^. The anomalous warming observed here has the opposite response with the potential to impact sea-ice production and melt, given the high sea-ice sensitivity to the stability of the seawater column^28^. This indicates a potential contribution to enhance thinning of the Larsen Ice Shelf in the Antarctic Peninsula^23–25^.

## Last Millennium Eruptions

The ocean response to LM eruptions is more evident than that for modern eruptions^2,7^, since historical eruptions are known to be much larger than the more recent ones^29,34^. The 1815 Tambora eruption, for example, loaded 109Tg of SO_2_ into the stratosphere^49^, which caused 1 °C decrease in global SST^2^. In contrast, the Mt. Pinatubo eruption in 1991 decreased the SST by 0.4 °C^2^, with a stratospheric input of sulfur-containing gases of 30Tg^49^. We therefore use CESM-LME results from 850 to 1850, to understand the physical mechanisms behind the Weddell Sea response to all major eruptions over the Last Millennium. Here major eruptions^34^ are those with an aerosol mass mixing ratio above 10^−8^; this leads to a set of seven eruptions (Table 1) located in the tropics and Southern Hemisphere. We considered both regions together, since both have a large impact on the southern extra-tropics with similar temperature response^34^. In addition, by considering the eruptions from both regions we include more events in the analysis (105 in total, see Methods), which increases the signal-to-noise ratio beyond what would be possible with either event type alone^33,34^. All anomalies shown for the LM are relative to the 850–1850 climatological mean^34^ only for the austral summer (DJF).

**Table 1:** Last Millennium selected large eruptions. The source region, eruption year^34^, name of the volcano and SO_2_ loading into the stratosphere^49^ of the seven volcanoes considered in this study^35,51^.

Source Region	Year	Volcano	SO_2_ Loading (Tg)
Tropical	1258	Samalas	257.91
	1284	—	54.69
	1809	—	53.74
	1815	Tambora	109.72
Southern	1275	—	63.72
	1341	—	31.14
	1452	Kuwae	137.59

**Figure 3:**
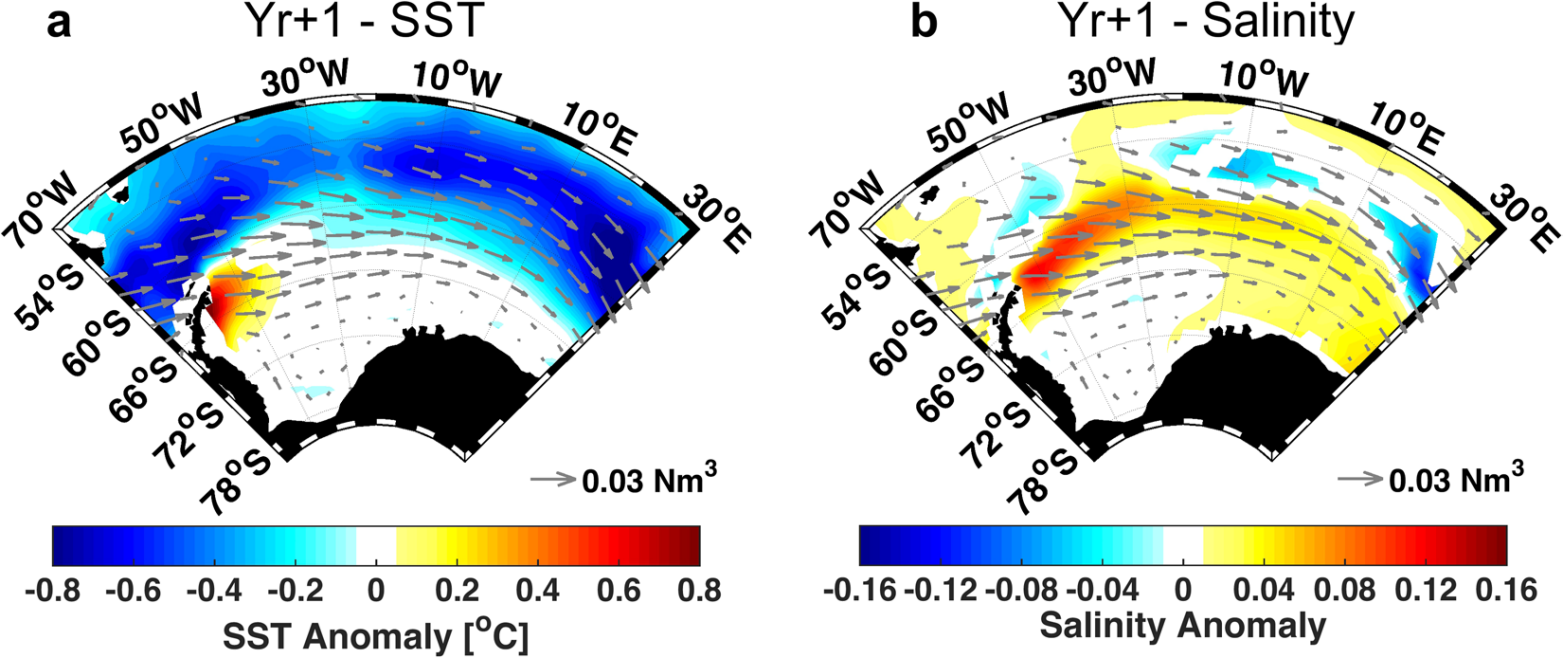
Atlantic Southern Ocean sector surface temperature and salinity anomalous response to LM eruptions (Yr + 1). DJF composite response to the selected eruptions only showing Yr + 1 for **(a)** SST [°C] and **(b)** Salinity anomalies. Vectors show the wind stress anomaly [N m^−2^]. For SST and Salinity only regions that are significant at 90% are shown.

In the composite evolution from Yr0 to Yr + 3 for SST and salinity (Supplementary Figs. S3 and S4), the greatest signal (significant at 90% level according to the Wilcoxon rank-sum test) is seen during the first year after the eruption (Yr + 1). The same anomalous warming pattern (here of ∼0.8 °C) near the Antarctic Peninsula in the Weddell Sea from results after Mt. Pinatubo eruption is observed for LM eruptions in the model results (Fig. 3a). At the same time, there is a positive salinity anomaly (∼0.16) off the northern Antarctic Peninsula that spreads eastward (Fig. 3b). The year of the largest response after LM eruptions is different than that after Mt. Pinatubo eruption. For the LM eruptions the greatest response is at Yr + 1, while for Mt. Pinatubo it is at Yr + 2. This difference may be explained by the size of the eruption, since most responses scale with the magnitude of the eruption^50,51^. The surface ocean response to LM eruptions may also be faster than for the Mt. Pinatubo one, because of their larger size^34,49^.

**Figure 4:**
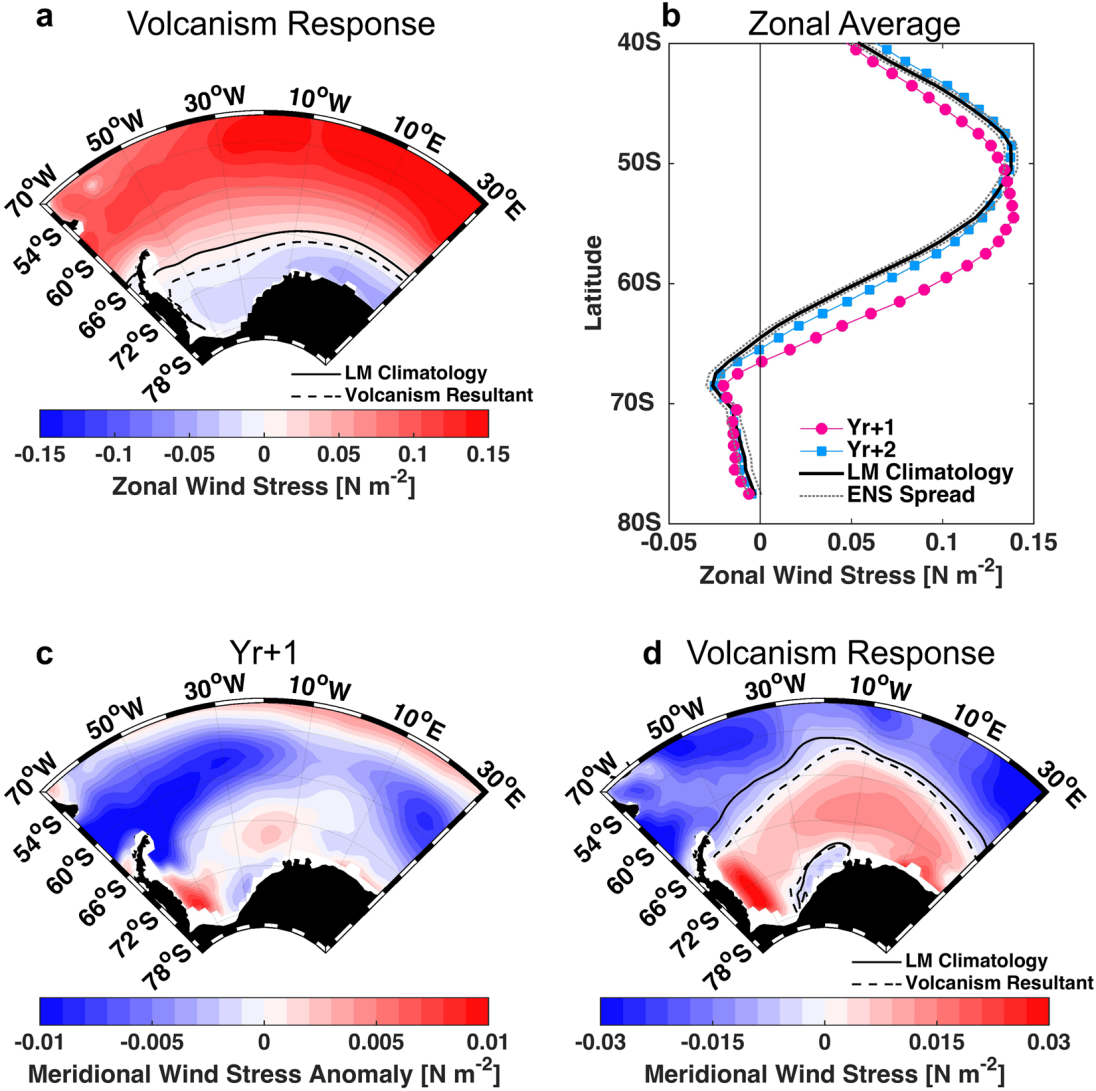
Atlantic Southern Ocean sector wind stress anomalous response to LM eruptions (Yr + 1). DJF composite response to the LM selected eruptions. **(a)** Zonal wind stress [N m^−2^], continuous line representing the zero contour for LM climatological mean and the dotted line the zero contour resultant after the year after the eruption. **(b)** Zonal average for zonal wind stress [N m^−2^] for the South Atlantic Ocean (70°W-30°E, 40°-80°S) for eruption subsequent years. **(c)** Meridional wind stress anomaly [N m^−2^] at Yr + 1. **(d)** The same as **(a)**, but for the meridional wind stress [N m^−2^].

The CESM-LME wind stress anomaly reveals an intensified westerly flow (represented by vectors in Fig. 3a,b), which is further investigated separately for each component (zonal and meridional). The zonal wind stress substantially changes at Yr + 1 (Fig. 4a). During the year after the eruption (Yr + 1), its southernmost extension has shifted southward compared to the climatology (dotted and continuous lines, respectively, in Fig. 4a). The southward shift starts at Yr0 (1.48° of latitude, Supplementary Fig. S5) and increases to 2.16° at Yr + 1. This shift is larger than the westerlies interannual variability during the LM (±1.67°). In addition, there is an intensification of ∼20% (0.03 N m^−2^). The same pattern of meridional shift and westerlies intensification was observed after the Mt. Pinatubo eruption (Fig. 2b). The westerlies southward shift and strengthening has been also shown by other authors^29,52,53^. It is related to the overall intensified equator-to-pole temperature gradient after a large eruption, caused by the aerosol absorption that causes warming in the tropical region of the lower-stratosphere^2^ and also related to the zonal flow-planetary wave interactions^52^. This is more evident for LM eruptions than for Mt. Pinatubo eruption, because the large internal climate variability of Mt. Pinatubo eruption could obscure this forced response^33,53^.

The meridional wind stress climatological pattern in the Weddell Sea is a northward flow that reverts direction in the northernmost portion of the Antarctic Peninsula (continuous line in Fig. 4d). Due to volcanic eruptions, the response in the meridional wind stress is a negative anomaly off the northern Antarctic Peninsula (Fig. 4c), which is sufficient to reverse the circulation in this region. The position of the zero meridional wind stress shifts southward after the eruption (dotted line in Fig. 4d).

The surface wind system changes help explain the anomalous warming near the Antarctic Peninsula in the Weddell Sea. The substantial wind stress field changes have an impact on the ocean surface currents (Supplementary Fig. S6). Zonal and meridional surface velocities anomalies present a 10% to ~14% increase in the flow, respectively, mainly in the southern extension of the ACC (Supplementary Fig. S6). After the eruption the currents near the Antarctic Peninsula are highly influenced by the flow at the southern end of the ACC. This means that the stronger ACC flow enhances mixing further south.

**Figure 5:**
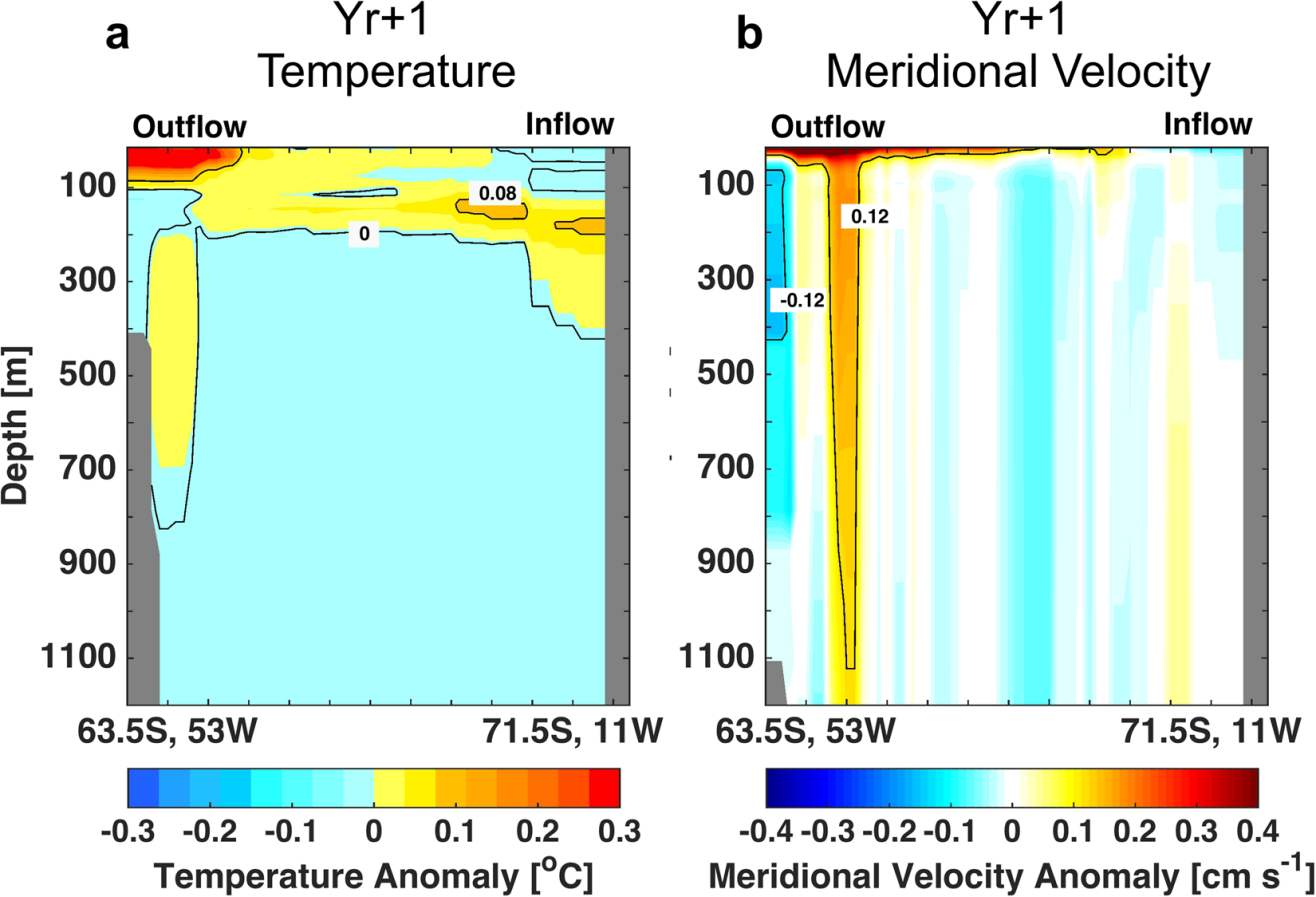
Depth profile composite anomalies in the year after LM eruptions (Yr + 1). **(a)** Potential temperature anomaly [°C]. **(b)** Meridional velocity anomaly [cm s^−1^]. Both only shown for DJF anomalies. The transect is at the same location of SR4*/*WOCE stations. The inflow and outflow regions are marked, respectively 71.5°S,11°W and 63.5°S,53°W.

To determine the response of the Weddell Sea below the surface we investigate CESM-LME results for the same location of the SR4 stations (Supplementary Fig. S7) from World Ocean Circulation Experiment (WOCE)^54^ that covers both the inflow and outflow regions of the Weddell Gyre (WG, marked in Fig. 5). The depth profile of temperature anomaly shows a small warming of 0.08 °C in the inflow region, around 200–300 m (Fig. 5a). We hypothesize that the warming could be caused by weakened convection^28^, but the mechanisms for this are not examined here in detail. At the same time, in the outflow region the positive anomaly is concentrated in layers shallower than 100 m (Fig. 5a).

The depth profile of the meridional velocity anomaly for the first year after the eruption (Fig. 5b) shows a negative anomaly at the subsurface outflow region of ~0.12 cm s^−1^, from ∼100 m to 400 m deep. This represents a decrease of 16% in the subsurface WG outflow. At the same time, at the surface and shifted eastward there is a positive anomaly of the same magnitude (~0.12 cm s^−1^), which is explained by the enhanced flow in the southern limit of the ACC (Supplementary Fig. S6).

## Mechanism for Surface Warming

**Figure 6:**
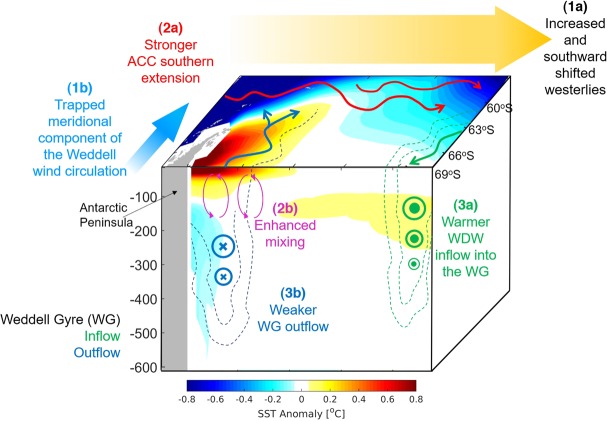
Schematics from the suggested mechanism in the Weddell Sea near the Antarctic Peninsula after a large eruption. The surface and vertical section in the location of SR4*/*WOCE stations represent the temperature anomaly for the year after the eruption (Yr + 1). The wind circulation is represented in yellow and blue thick arrows. The red arrows represent the ACC southern extension. The inflow and outflow of the WG are in green and blue, respectively. In the surface the WG is represented by the thin arrows and along the depth as dotted contours with notations for vectors coming in and out of the vertical plane.

Our results indicate that the surface temperature off the Antarctic Peninsula is not primarily responding directly to the radiative forcing effect from volcanism, but is driven by the delayed response of ocean dynamics in the first year after the eruption (Yr + 1). We believe that the dynamical response is as follows and schematically represented in Fig. 6, with respect to:

1. ***Winds***: The westerlies become stronger and their southernmost extension also shifts south into most of the Weddell Sea(Fig. 6-1a). At the same time, along the outflow region in the Weddell Sea near the Antarctic Peninsula, the northward winds are displaced southward (Fig. 6-1b).
2. ***Surface Circulation***: As a response to the wind changes, the southern limit of the ACC becomes stronger (Fig. 6–2a), enhancing mixing at the Weddell Sea outflow region (Fig. 6–2b).
3. ***Subsurface Circulation***: At the subsurface layer, ~400 m depth, the inflow region is somewhat warmer in 0.08 °C (Fig. 6–3a). At the same time that the WG outflow is weakened (Fig. 6–3b). This means that the subsurface water that is normally exported out of the WG gets trapped in the region near the Antarctic Peninsula.
4. The enhanced mixing and the subsurface water accumulating in the outflow region, due to weaker outflow, brings up warmer and saltier subsurface waters, as the Warm Deep Water (WDW, Supplementary Fig. S7). This water gets trapped at the surface outflow region because of the wind changes.

Our results show that Weddell Sea dynamics are the primary driver of warming off the northern tip of the Antarctic Peninsula following major volcanic eruptions. This warming is a response of the WG to the southward shift and strengthening of the Antarctic westerlies, together with the southward wind anomaly at the WG outflow region. In this region, warm and salty WDW waters, through convective mixing, reaches surface layers. Because of the anomalous wind response, the outflow is weakened and trapped leading to the positive SST anomaly detected.

## Final Considerations

We quantified the SST perturbation caused by the Mt. Pinatubo 1991 eruption, which showed an anomalous warming response near the Antarctic Peninsula in the Weddell Sea at Yr + 2. Such response was not expected given that the known impact of volcanic eruptions is an overall cooling^4,5,7^. The physical mechanism responsible for this surface warming was investigated considering the LM (850–1850) largest eruptions. Even though the LM eruptions are known to be much stronger than Mt. Pinatubo and have different intrinsic characteristics (i.e. intensity), we observed the same robust anomalous warming near the Antarctic Peninsula in the Weddell Sea. In addition, any eruptions occurring in the future could be as strong as the LM ones, and given the robust warming response in the Weddell Sea, we anticipate that such future large eruptions would once again generate significant warming in the region.

Composite ensemble average for LM eruptions showed ∼0.8 °C SST increase accompanied by positive salinity anomaly (~0.16) developed near the Antarctic Peninsula in the Weddell Sea during the first year after the eruption (Yr + 1). The composite analysis for the simulated LM eruptions presented the same sea surface temperature pattern as after the Mt. Pinatubo eruption (averages from 0.2 to 0.85 °C for different data sets), however it is the largest at Yr + 1, while the Mt. Pinatubo largest response is at Yr + 2. The different year of the largest sea surface response is related to the size of the analyzed eruptions; LM eruptions are much larger than more recent ones^2,34^, therefore the ocean response is faster.

We suggest within the confines of the CESM-LME model^55^ that the anomalous warm SST response to large volcanic eruptions in the Weddell Sea is due to changes in the ocean dynamics, rather than to the direct radiative forcing. During the first year after the eruption (Yr + 1), a strengthening of 0.03 N m^−2^ of the prevailing westerlies was observed, which was accompanied by a southward shift of ∼2° of its southernmost extension. At the same time, the meridional component of the wind stress near the northern Antarctic Peninsula changes direction from northward to southward. As a response to the wind changes, the ACC intensifies at its southern limit in about 10%, enhancing mixing off the northern Antarctic Peninsula, together with a ∼16% weakening of the WG subsurface outflow down to ~400 m deep. Consequently, some of the water that was normally exported out of the WG is trapped at the subsurface region near the Antarctic Peninsula in the Weddell Sea. The subsurface water (i.e., WDW), that is warmer and saltier than the surface water, is brought up to the surface by the enhanced mixing. Because of the anomalous wind this water gets trapped in the Weddell Sea surface outflow region near the Antarctic Peninsula.

The results shown here underscore the concern about ocean-induced thinning of ice shelves, that has been accelerating over the past years^26^. The warming response of the Weddell Sea off the Antarctic Peninsula to large volcanic eruptions could impact the ongoing thinning of the Larsen Ice Shelf, through basal melting^23,26^ and the intrusion of warm ocean waters^18^. Considering that Earth System Models suggest that the climate response to eruptions will be amplified in the future^17^ due to projected increases in ocean stratification, further warming in the Weddell Sea due to volcanic eruptions could enhance the probability of sudden ice shelf collapse. Therefore, despite the global cooling associated with volcanic eruptions, the dynamical response actually enhances the regional response to anthropogenic warming in the Antarctic Peninsula, which highlights the Southern Ocean’s vulnerability^18–20,25^.

## Methods

### Data sets and Mt. Pinatubo eruption

Volcanically induced changes in the Southern Ocean Atlantic sector are examined after the 1991 Mt. Pinatubo eruption. For that we restrict the analysis to 1980–2005 period comparing available observational data sets and output from the Last Millennium Ensemble experiment from the Community Earth System Model^33^ (CESM-LME, described in the next section). For the Mt. Pinatubo response all anomalies are calculated relative to the 1980–2005 mean. The anomaly time series is de-trended over the same period, as a way to minimize CO_2_-induced long-term trend that could skew the results. For the same period (1980–2005), we also examined the average of the CESM-LME ensemble containing only the volcanic forcing, that allows us to isolate the volcanic signal on the SST.

For all the analysis we considered only the austral summer (DJF), because of the smaller sea-ice extension in the Weddell Sea during this season. In addition, the maximum response to volcanic activity is in the summer for both hemispheres, which is probably a result of shallow mixed layers at this time of the year, that respond more rapidly to changes in the heat flux^4^. This means that in the Mt. Pinatubo analysis the austral summer of the year of the eruption (1991) consider December from this year and January and February from the next year (1992). For all the other years we used the same approach.

We utilized SST data from the Extended Reconstructed Sea Surface Temperature version 5 (ERSSTv5)^30^ from the National Oceanic and Atmospheric Administration (NOAA). The product has a spatial resolution of 2° and covers January 1854 up to 2015. Also, the Hadley Centre Sea Ice and Sea Surface Temperature (HadISST) data set^31^, with 1° latitude-longitude grid from 1870 to 2017. Other data sets used are oceanic reanalysis such as the Simple Ocean Data Assimilation (SODA) reanalysis version 3.4.1^32,36^, that uses an ocean general circulation model to assimilate available *in situ* temperature and salinity profiles, also data from satellite, forced by European Centre for Medium-Range Weather Forecasts (ECMWF) ERA-Interim^56^. The product is a gridded data set of monthly values from 1980 to 2015, at 0.5° horizontal resolution^32^. The Ocean Reanalysis System 4 (ORAS4) from ECMWF is also compared, with spatial resolution of 1° and monthly data from 1958 to 2017^11^.

### Model configuration and Last Millennium eruptions

All results from the Last Millennium (LM, 850–1850) are obtained from the CESM-LME^33^. The model configuration used the Community Earth System Model version 1.1 with the Community Atmosphere Model version 5^57,58^. The spatial resolution of the atmosphere and land components is ∼2° and the ocean and sea-ice components use ∼1° spatial resolution and 60 layers as ocean vertical resolution^33^. There is a total of 30 ensemble members of forced runs for the period between 850 and 2005, which are a combination of full forcing runs evolving in time and runs considering of each forcing individually with all other forcings fixed at values from the year 850. These are volcanic only, solar^59^, land use^60,61^, greenhouse gas, ozone-aerosol^62^ and orbital only^63^. The ensemble approach allows improved estimate of the uncertainty from internal variability and modeling process^37^. The volcanic eruptions are one of the most important forcings from the LM^64^, CESM-LME uses eruptions from reconstruction version 1^49^. This reconstruction^49^ accounts a total of 53 ice cores (32 from the Arctic and 22 from Antarctica), composing a comprehensive stratospheric volcanic sulfate mass loading for the past 1500 years^49^.

For the LM eruptions analysis, we consider the output from 10 full forcing and 5 volcanic-only ensemble members. This analysis has been restricted to the period 850–1850, to exclude any CO_2_-induced trends in the full forcing runs. The ensemble average is used to reduce the internal variability^65^. All anomalies presented in the LM eruptions analysis are calculated relative to the 850–1850 mean. The eruptions were selected according to a previous study that used the same model outputs^34^. The authors only selected eruptions with a peak aerosol mass mixing ratio greater than 10^−8^ and classified them in regions^34^. Here we selected only the tropical and southern eruptions, since both regions have shown to influence the southern extra-tropics ocean^34^.

All seven selected eruptions were averaged together. The year of the eruption is referred as Yr0 in the composite analysis, which means that all 7 eruptions from the 15 ensemble members were averaged. The composite evolution starts in the year before the eruption (Yr-1) and includes the 6 subsequent years (Yr + 1 to Yr + 6). As in the Mt. Pinatubo section, we only analyze austral summer (DJF) results, for that we considered the average of December, from the year of the eruption, January and February, from the subsequent year.

The significance levels presented are determined according to the Wilcoxon Rank Sum test at 90%^34^. The set of the composite years (7 eruptions from 15 ensemble members, total of 105 events) are compared with the full time series from all ensemble members. This is the nonparametric version of the t-test, which only makes the assumption of independence between the time series and equal variance, but not that the data have a known distribution^66^. We use the closest model grid points from the original historical stations from the SR4*/*WOCE (SR4 transect from World Ocean Circulation Experiment)^54^, which captures the subsurface inflow and outflow of Weddell circulation.

## Supplementary information

Supplementary Information

## Data Availability

The datasets generated during and/or analysed during the current study are available in the Earth System Grid repository [https://www.earthsystemgrid.org]. Other data sets (SODA, ERSST, HadISST and ORAS4) are available in Climate Data Guide repository [https://climatedataguide.ucar.edu/].
