# Supplementary material for: Volcanically Triggered Ocean Warming Near the Antarctic Peninsula
Source: Sci Rep. 2019 Jul 1;9:9462. doi: 10.1038/s41598-019-45190-3 (PMC6603043; doi:10.1038/s41598-019-45190-3)
Supplement: Supplementary file 1 — Supplementary Information [file 41598_2019_45190_MOESM1_ESM.pdf]

Volcanically Triggered Ocean Warming Near the Antarctic Peninsula

Verona, L. S.[*,1] ; Wainer, I.[1] & Stevenson, S.[2]

[1]*Instituto Oceanografico, Universidade de Sao Paulo (IO/USP) Sao Paulo, SP, Brazil.*

[2]*Bren School of Environmental Science & Management, University of California, Santa Barbara, CA.*

[*]*e-mail:* verona.laura@usp.br

Supplementary Information

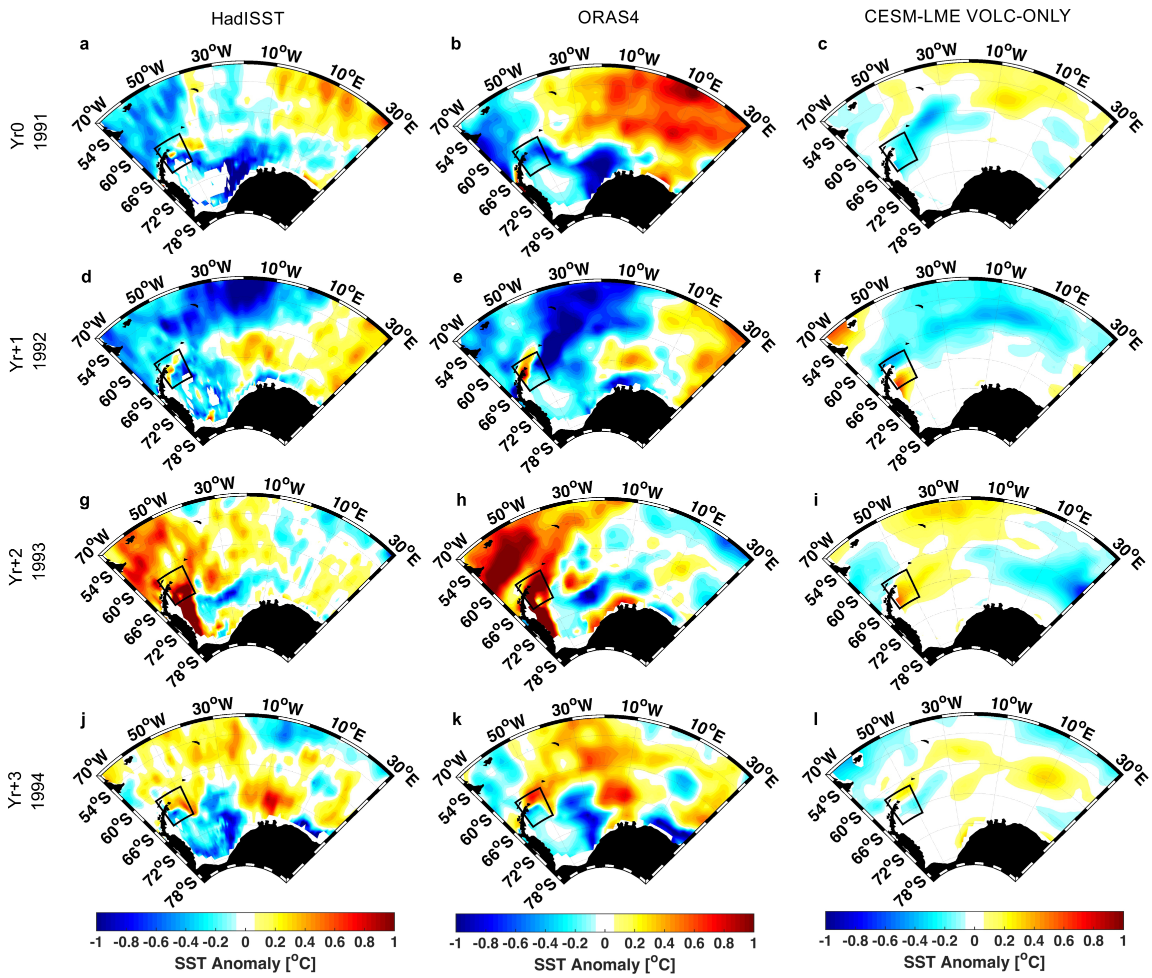

Figure S1: **DJF SST [°C] after Mt. Pinatubo eruption** | Left-hand column for HadISST [**(a), (d), (g), (j)**], central column for ORAS4 [**(b), (e), (h), (k)**] and right-hand column for CESM-LME Volvanic-Only ensemble average [**(c), (f), (i), (l)**]. Each row represent different years from 1991 (Yr0, the eruption year) to 1994 (Yr+3). The delimited area marks the region off the Antarctic Peninsula (60°-48°W, 61°-67°S).

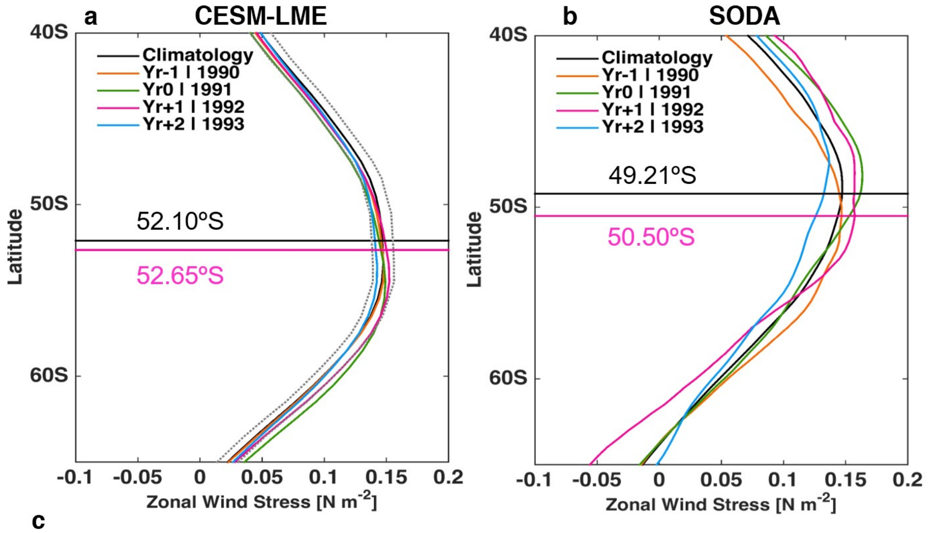

c

Mt. Pinatubo Eruption				
Data Sets	Year	Max TAUX	Latitude	Shift
CESM-LME	CLIM (1980-2005)	0.1475	52.10°S	-
	Yr-1 | 1990	0.1479	52.50°S	-0.402°
	Yr0 | 1991	0.1492	53.11°S	-1.005°
	Yr+1 | 1992	0.1524	52.65°S	-0.545°
	Yr+2 | 1993	0.1425	52.17°S	-0.074°
SODA	CLIM (1980-2005)	0.1473	49.21°S	-
	Yr-1 | 1990	0.1467	50.14°S	-0.932°
	Yr0 | 1991	0.1634	48.60°S	0.602°
	Yr+1 | 1992	0.1574	50.50°S	-1.294°
	Yr+2 | 1993	0.1367	48.38°S	0.826°

Figure S2: **Zonal wind stress [N m^{-2}] after Mt. Pinatubo eruption** | South Atlantic Ocean average (70°W-30°E, 40°-80°S) for climatological mean (1980-2005), Yr-1 (1990), Yr0 (1991), Yr+1 (1992) and Yr+2 (1993) of **(a)** CESM-LME ensemble average and **(b)** SODA. Horizontal lines indicate the associated maximum zonal wind stress position calculated from the weighted averaged method for the climatology (black line) and Yr+1 (1993, magenta line). **(c)** Table specifications of the maximum zonal wind stress magnitude, its latitudinal position and the shift from the climatological position for both data sets.

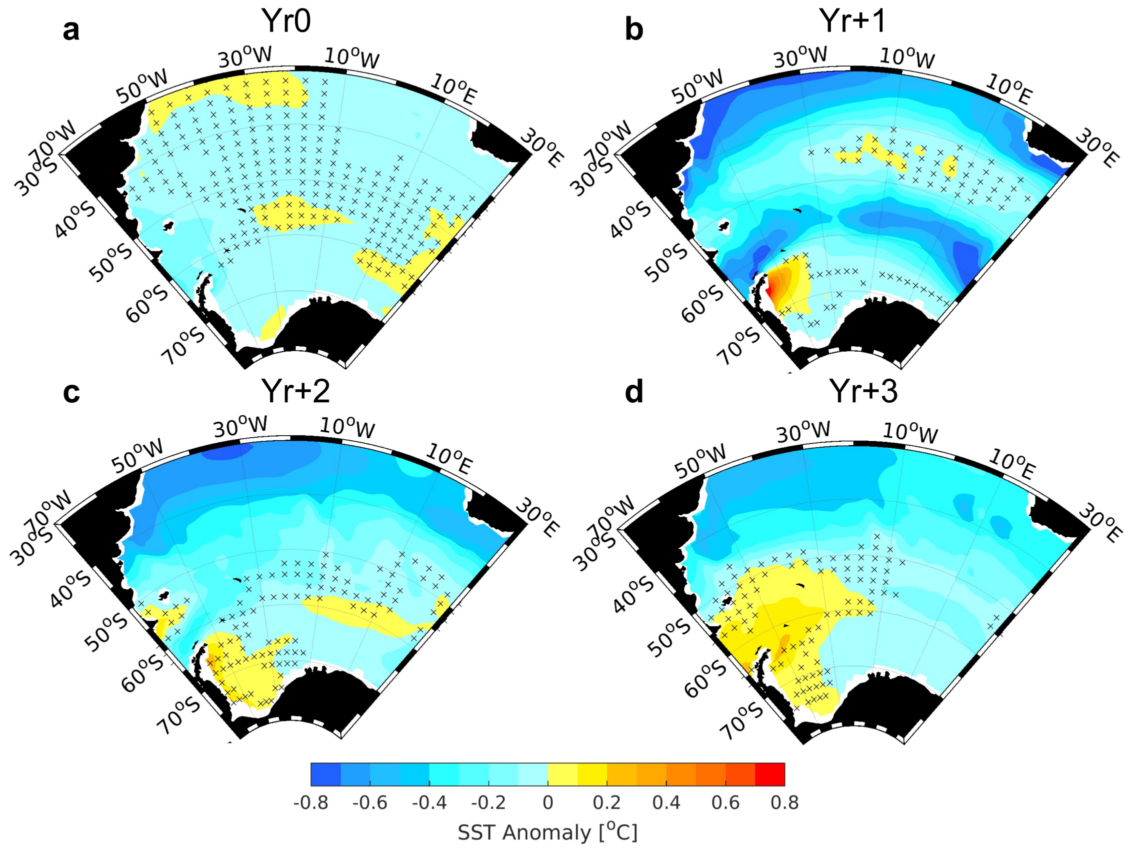

Figure S3: **Last Millennium DJF SST anomaly composites from Yr0 to Yr+3** | SST [°C] response to selected eruptions for **(a)** Yr0, **(b)** Yr+1,**(c)** Yr+2 and **(d)** Yr+3. Significance levels are determined according to the Wilcoxon Rank Sum test, values that are insignificant at 90% are marked with crosses.

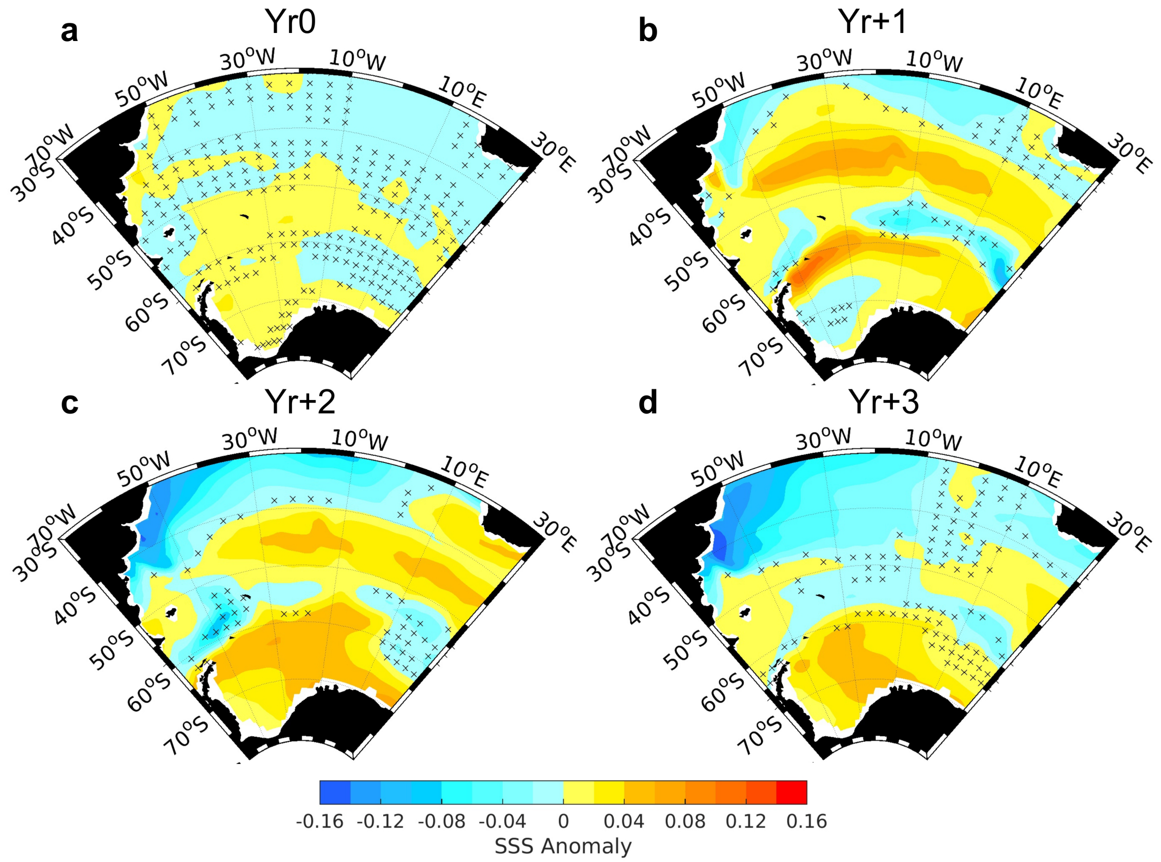

Figure S4: **Last Millennium DJF salinity anomaly composites from Yr0 to Yr+3 |** Salinity response to selected eruptions for **(a)** Yr0, **(b)** Yr+1,**(c)** Yr+2 and **(d)** Yr+3. Significance levels are determined according to the Wilcoxon Rank Sum test, values that are insignificant at 90% are marked with crosses.

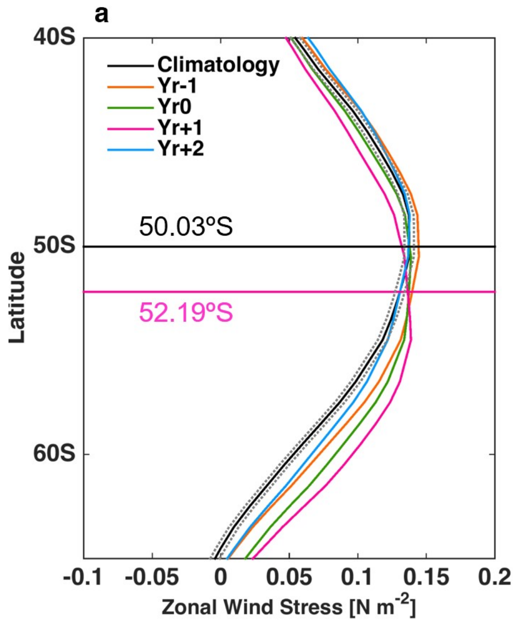

b

Last Millennium Eruptions			
Year	Max TAUX	Latitude	Shift
CLIM (850-1850)	0.1375	50.03°S	
Yr-1 | 1990	0.1446	50.52°S	-0.497°
Yr0 | 1991	0.1388	51.50°S	-1.477°
Yr+1 | 1992	0.1388	52.19°S	-2.160°
Yr+2 | 1993	0.1378	50.06°S	-0.034°

Figure S5: **Zonal wind stress [N m^{-2}] response to LM eruptions** | South Atlantic Ocean average (70°W-30°E, 40°-80°S) for ensemble climatological mean (850-1850) and composites for Yr-1, Yr0, Yr+1 and Yr+2 of **(a)** CESM-LME eruptions of the LM. Horizontal lines indicate the associated maximum zonal wind stress position calculated from the weighted averaged method for the climatology (black line) and Yr+1 (1993, magenta line). **(b)** Table specifications of the maximum zonal wind stress magnitude, its latitudinal position and the shift from the climatological position.

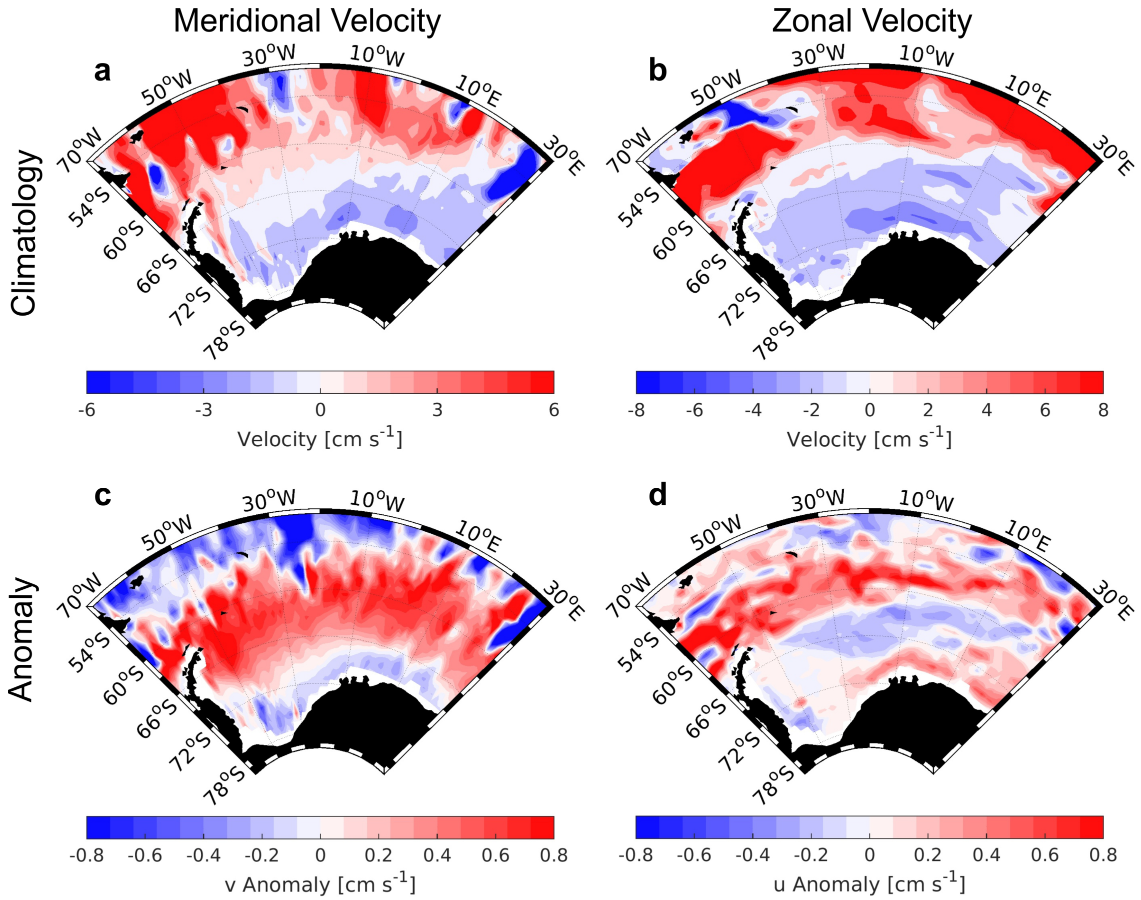

Figure S6: **DJF Surface ocean velocities** | [**(a)**, **(b)**] Climatological mean field for the Last Millennium velocity components, meridional and zonal [cm s^{-1}] respectively. [**(c)**, **(d)**] Associated anomaly in the meridional and zonal components [cm s^{-1}] due to volcanism during the year after the eruption (Yr+1).

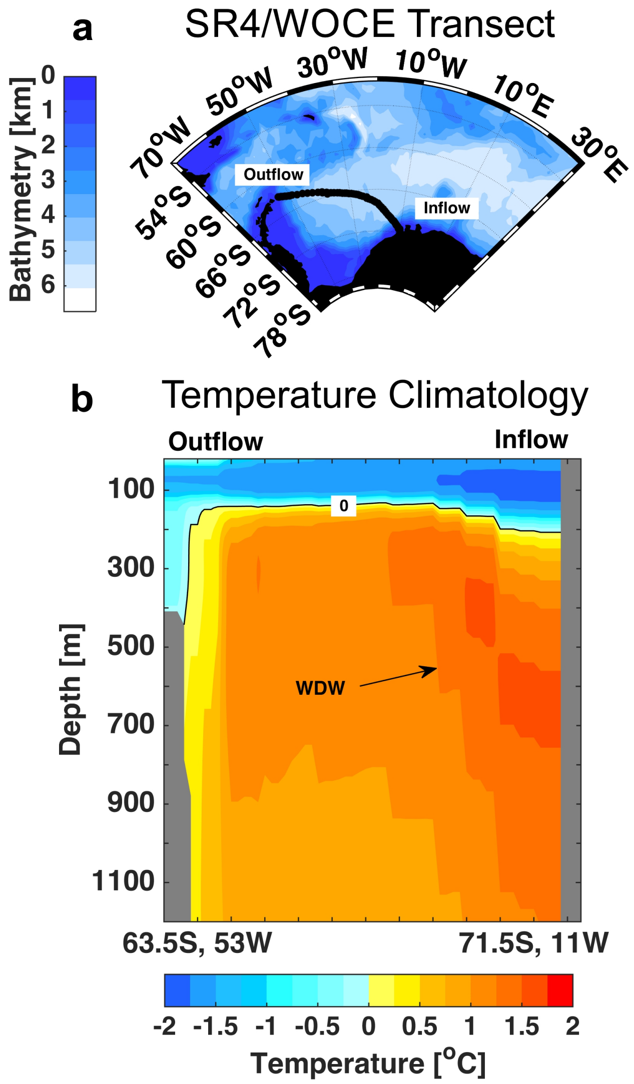

Figure S7: **SR4/WOCE transect representation in LM eruptions** | **(a)** Bathymetry map for Atlantic sector of the Southern Ocean showing the location of SR4/WOCE transect in black, with indications of the Weddell Gyre inflow and outflow regions. **(b)** Climatological Last Millennium mean of the potential temperature [°C] profile for the location of the SR4/WOCE transect. The inflow and outflow regions are marked, respectively 71.5°S,11°W and 63.5°S,53°W. The Warm Deep Water(WDW)core is pointed.
